# Severe Cholestatic Drug-Induced Liver Injury With Cephalosporin Use

**DOI:** 10.7759/cureus.32262

**Published:** 2022-12-06

**Authors:** Kevin Yang, Tiberiu Moga, Naren S Nallapeta, Robert Duve, Clive J Miranda, Mayada Ismail, Thomas Mahl

**Affiliations:** 1 Internal Medicine, University at Buffalo, Buffalo, USA; 2 Gastroenterology, University at Buffalo, Buffalo, USA; 3 Department of Medicine, State University of New York, Buffalo, USA; 4 Department of Gastroenterology and Hepatology, Erie County Medical Center’s (ECMC), Buffalo, USA

**Keywords:** liver injury biomarkers, cholestatic liver injury, cephalosporins, drug-induced acute liver failure, drug-induced liver injury (dili)

## Abstract

Drug-induced liver injury (DILI) is a phenomenon that occurs with nearly all classes of medications. Cholestatic DILI represents a fraction of these cases and can present as bland cholestasis, cholestatic hepatitis, secondary sclerosis cholangitis, and vanishing bile duct syndrome. Risk factors have been identified for cholestatic DILI, including older age, genetic determinants, and certain medications such as amoxicillin-clavulanate. Here, we describe a complicated case of severe cholestatic DILI secondary to cephalosporin use. A 27-year-old female presented to the hospital initially with fever and abdominal pain for four weeks after an emergency C-section for pre-eclampsia and hemolysis, elevated liver enzymes, lowered platelets (HELLP) syndrome. She was found to have a retroperitoneal abscess and underwent bilateral drain placement. She was initially started on cefazolin, and then coverage was broadened to cefepime. Shortly after, alkaline phosphatase (ALP) rose and peaked at 3498 IU/L, with aspartate aminotransferase (AST) and alanine transaminase (ALT) elevated at 274 IU/L and 122 IU/L, respectively. Extensive testing for secondary causes and a liver biopsy were consistent with DILI. Liver enzymes down-trended with the cessation of cefepime. This case report highlights that prompt recognition of the culprit medication is paramount to recovering normal liver function.

## Introduction

According to the American Drug-Induced Liver Injury (DILI) Network, the most common drug classes implicated in DILI were antibiotics, representing 45.4% of total cases, followed by herbal and dietary supplements, cardiovascular agents, and CNS agents. The most common individual drugs identified were amoxicillin-clavulanate, isoniazid, and nitrofurantoin. Cefazolin ranked sixth, ceftriaxone was identified in a single patient, and cefepime was not mentioned at all [1,2]. Cephalosporins present with an acute cholestatic pattern, characterized as either bland cholestasis or cholestatic hepatitis. Bland cholestasis results from abnormal biliary secretion and is not associated with hepatocellular damage, whereas cholestatic hepatitis is accompanied by an inflammatory cell infiltrate, degenerative changes, and possible hepatocellular necrosis [3]. Medications can also cause chronic cholestasis through either obliteration of bile ducts or extrahepatic biliary obstruction [4].

## Case presentation

A 27-year-old female presented to the hospital initially with fever, abdominal pain, nausea, and vomiting for four weeks after an emergency C-section for pre-eclampsia and hemolysis, elevated liver enzymes, lowered platelets (HELLP) syndrome. She was also treated for severe hypertriglyceridemia and pancreatitis with multi-organ failure and acute respiratory distress syndrome. Vital signs at the presentation were BP 80/50, HR 130, and RR 21. The physical exam showed a distended abdomen with mild, diffuse tenderness to palpation throughout. WBC was 2.7, Hgb was 9.1 g/dL, and platelets were 373. Basic metabolic panel (BMP) showed Na 122, acute kidney injury (AKI) with a creatinine of 2.4 mg/dL, and elevated anion-gap metabolic acidosis with a lactate of 7.6 mmol/L. Initial liver enzymes were: ALP 122 IU/L (upper limits of normal [ULN] < 129 IU/L), AST 20 IU/L (ULN < 38 IU/L), and ALT 28 IU/L (ULN < 42 IU/L). A CT scan of the abdomen and pelvis confirmed large-volume ascites, hepatic steatosis with hepatosplenomegaly, and ill-defined peripancreatic fluid.

**Figure 1:**
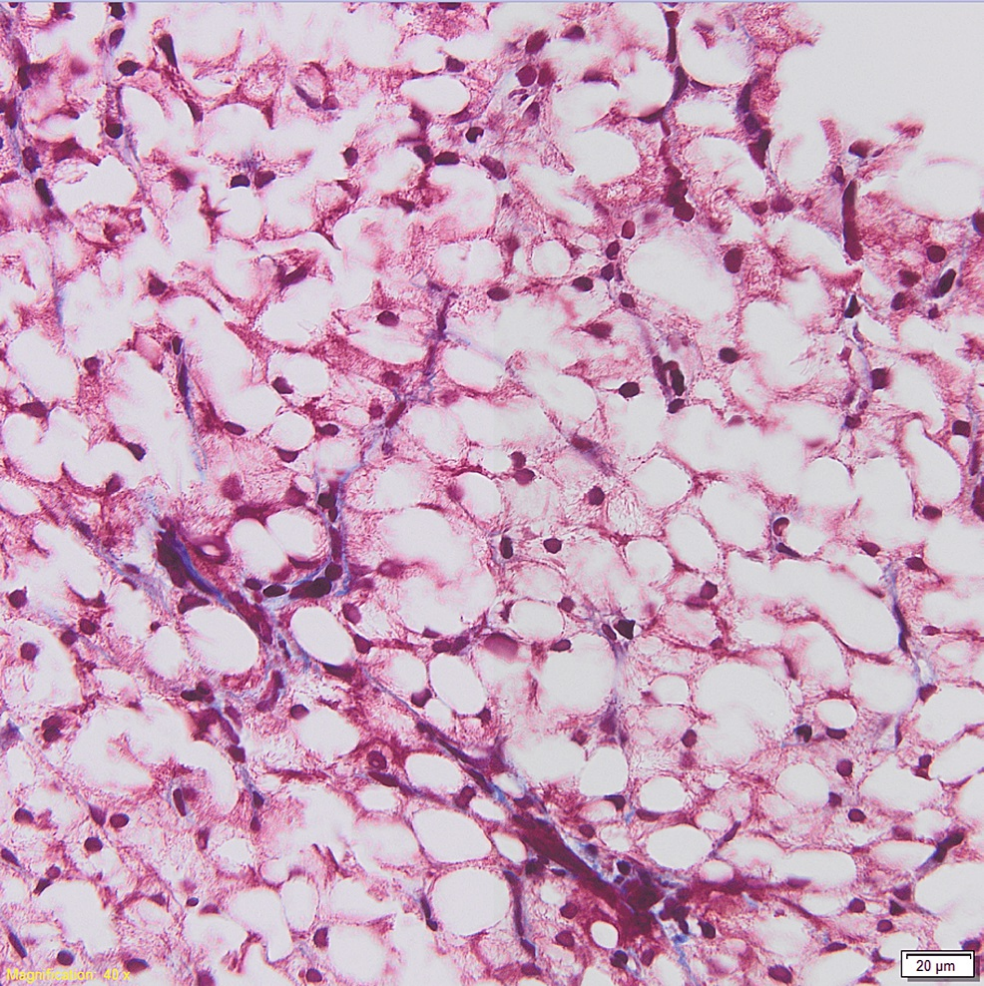
Trichrome slide of macrovesicular changes in liver

**Figure 2:**
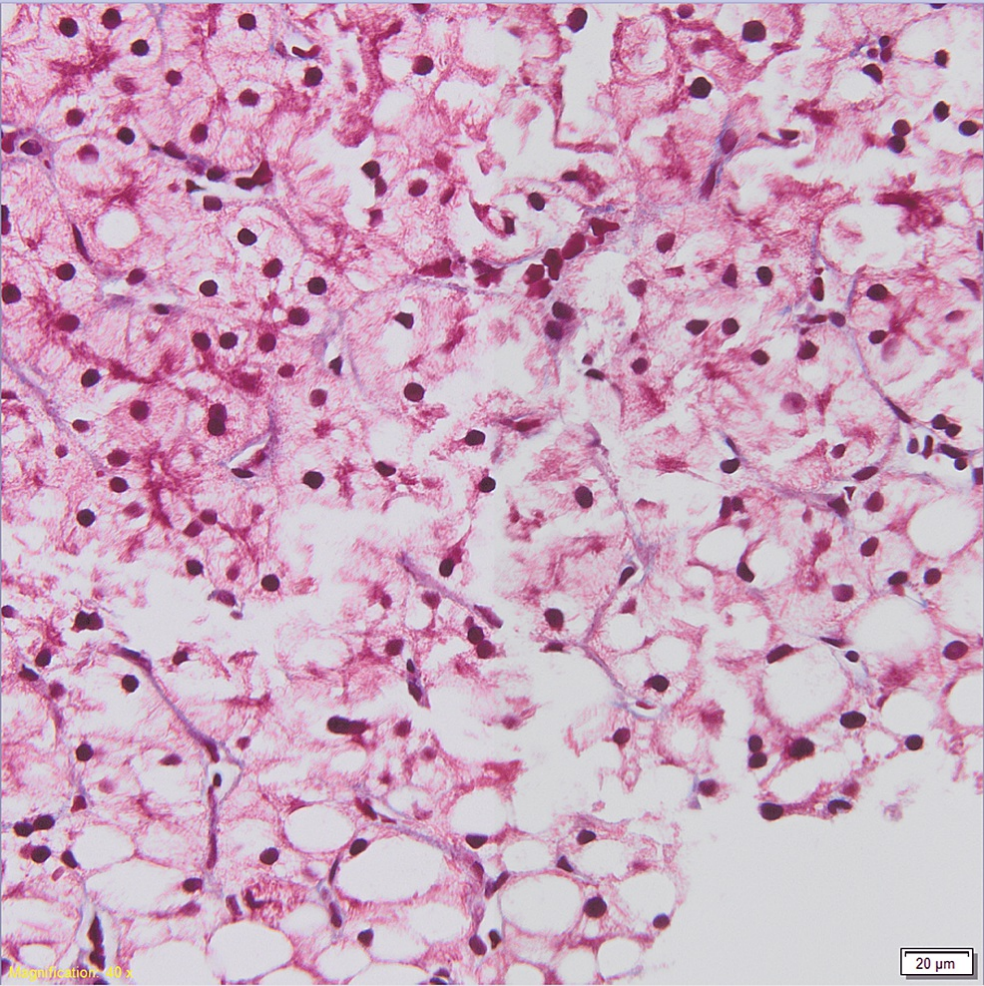
Trichrome slide of macrovesicular and microvesicular changes in liver

She had surgical debridement of a retroperitoneal abscess with bilateral drain placement. She started long-term antibiotic therapy for abdominal sepsis due to Escherichia coli with cefazolin. This was later changed to ceftriaxone and then cefepime. She was seen by gastroenterology initially for nausea, vomiting, and ascites (attributed to pancreatitis and secondary bacterial peritonitis). Esophagogastroduodenoscopy (EGD) during her first week of admission did not identify a cause of nausea and vomiting. Approximately one month into her hospitalization, ALP started rising and peaked at 3498 IU/L. The gamma glutamyltransferase (GGT) level was 1000 IU/L (ULN < 36 IU/L). AST and ALT were elevated to 274 IU/L and 122 IU/L, respectively. Differential diagnoses at that time included drug-mediated injury, viral hepatitis, autoimmune disease, ischemia or shock, and infection. Upon review of medications, we noted her prolonged treatment with cephalosporins. The toxicology screen was negative. Extensive testing for viral (hepatitis A virus [HAV], hepatitis C virus [HCV], herpes simplex virus [HSV], cytomegalovirus [CMV], and Epstein-Barr virus [EBV]) and autoimmune hepatitis was negative. Serology showed immunity to HBV. Ultrasound of the liver with doppler studies was unremarkable. A liver biopsy showed marked macrovesicular steatosis as well as microvesicular changes (Figures 1-2). No steatohepatitis, lobular inflammation, or apoptosis were present. The findings were compatible with drug-induced liver injury.

Cefepime was switched to ciprofloxacin, and her symptoms and liver function tests began to improve. After six weeks, her AST and ALT normalized, and her ALP declined to 802 IU/L. She was discharged to a medical rehabilitation unit for physical therapy. Table 1 compares the liver enzymes when ALP peaked with those after six weeks of withdrawing cephalosporins.

**Table 1:** Liver enzymes at peak alkaline phosphatase and six weeks after stopping cephalosporins ALT: alanine transaminase, ALP: alkaline phosphatase, AST: aspartate aminotransferase.

	At peak ALP Level	6 weeks
AST (ULN <38 IU/L)	112 IU/L	14 IU/L
ALT (ULN <42 IU/L)	31 IU/L	32 IU/L
ALP (ULN <129 IU/L)	3498 IU/L	802 IU/L
Bilirubin (ULN <0.3 mg/dL)	0.5 mg/dL	0.4 mg/dL

## Discussion

Cholestatic DILI is often challenging to diagnose and is often a diagnosis of exclusion. Among medications that are known to cause DILI, cephalosporins comprise approximately 1-2% of cases in prospective studies. These are often case reports, and generally, cephalosporins are widely used and well tolerated [5]. Cephalosporin-induced cholestatic DILI can result in acute or chronic liver injury. Although most cases of DILI resolve after cessation of the drug, some cases may even progress to cirrhosis. In one study, of the 1212 patients reviewed between 2004 and 2012, 19 of the 1019 DILI cases were attributed to cefazolin. This represented only 2% of cases [6]. Other cephalosporins, such as ceftriaxone and cefepime, are even less commonly documented. These patients suffered from moderate to severe liver injury, with a mixed or cholestatic pattern of hepatocellular injury.

Early recognition of cholestatic DILI is crucial, as there is no specific therapy. Treatment consists of withdrawing the suspected drug, avoiding drug rechallenge, and treatment of symptoms [7]. Patients with cholestatic DILI can present with nonspecific symptoms, including pruritis, malaise, abdominal pain, nausea, and encephalopathy. Some patients are asymptomatic. Physical exam findings may include jaundice, fever, rash, and other signs of acute liver failure [8]. Establishing a timeline regarding the onset of symptoms with respect to drug exposure can be helpful, as there are no reliable serologic markers to diagnose DILI. The most common method to assess causality between liver injury and the suspected drug is the Roussel-Uclaf Causality Assessment Method (RUCAM), which takes into account chronologic and clinical data, including exact exposure time, development of liver tests after cessation, documented hepatotoxicity, and the exclusion of competing etiologies [9]. However, the reliability of this model has been challenged and is confounded by the lack of a gold standard test for DILI.

Laboratory workup should include a complete metabolic panel, including liver function tests, a coagulation panel, a complete blood count, viral and autoimmune serologies, a toxicology screen, and ultrasound imaging. There is no established indication for a liver biopsy, although this is often performed to determine the severity and type of liver injury [10,11]. In our patient, a full workup of her acute liver injury highly suggested cephalosporins as the culprit. Regardless, prompt recognition of cholestatic DILI and cessation of the drug response are necessary to minimize long-term adverse effects.

## Conclusions

This case report highlights the importance of recognizing cephalosporins other than cefazolin as potential causes of DILI. We described a severe case of cholestatic DILI, as evidenced by the pattern of liver enzyme elevation. The rise and fall of these enzymes closely paralleled the initiation and cessation of cefepime in our patients. A thorough workup ruled out other secondary causes, and a liver biopsy confirmed our suspicion of DILI. While cephalosporins are still appropriately cornerstones of antibiotic coverage, it is imperative to be mindful of their potential for hepatotoxicity.
